# Investigating function roles of hypothetical proteins encoded by the *Mycobacterium tuberculosis* H37Rv genome

**DOI:** 10.1186/s12864-019-5746-6

**Published:** 2019-05-21

**Authors:** Zhiyuan Yang, Xi Zeng, Stephen Kwok-Wing Tsui

**Affiliations:** 1 College of Life Information Science & Instrument Engineering, Hangzhou Dianzi University, Hangzhou, 310018 China; 2 School of Biomedical Sciences, The Chinese University of Hong Kong, Shatin, N.T Hong Kong SAR; 3 Hong Kong Bioinformatics Centre, The Chinese University of Hong Kong, Shatin, N.T Hong Kong SAR; 4 Centre for Microbial Genomics and Proteomics, The Chinese University of Hong Kong, Shatin, N.T Hong Kong SAR

**Keywords:** *Mycobacterium tuberculosis*, Drug target, Virulence factor, Bioinformatics

## Abstract

**Background:**

*Mycobacterium tuberculosis* (MTB) is a common bacterium causing tuberculosis and remains a major pathogen for mortality. Although the MTB genome has been extensively explored for two decades, the functions of 27% (1051/3906) of encoded proteins have yet to be determined and these proteins are annotated as hypothetical proteins.

**Methods:**

We assigned functions to these hypothetical proteins using SSEalign, a newly designed algorithm utilizing structural information. A set of rigorous criteria was applied to these annotations in order to examine whether they were supported by each parameter. Virulence factors and potential drug targets were also screened among the annotated proteins.

**Results:**

For 78% (823/1051) of the hypothetical proteins, we could identify homologs in *Escherichia coli* and *Salmonella typhimurium* by using SSEalign. Functional classification analysis indicated that 62.2% (512/823) of these annotated proteins were enzymes with catalytic activities and most of these annotations were supported by at least two other independent parameters. A relatively high proportion of transporter was identified in MTB genome, indicating the potential frequent transportation of frequent absorbing essential metabolites and excreting toxic materials in MTB. Twelve virulence factors and ten vaccine candidates were identified within these MTB hypothetical proteins, including two genes (rpoS and pspA) related to stress response to the host immune system. Furthermore, we have identified six novel drug target candidates among our annotated proteins, including Rv0817 and Rv2927c, which could be used for treating MTB infection.

**Conclusions:**

Our annotation of the MTB hypothetical proteins will probably serve as a useful dataset for future MTB studies.

**Electronic supplementary material:**

The online version of this article (10.1186/s12864-019-5746-6) contains supplementary material, which is available to authorized users.

## Background

Tuberculosis (TB) remains a major global health problem and represents a great challenge in various regions in the world [1]. In 2012, the World Health Organization (WHO) estimated that 8.6 million people developed TB and 1.3 million people died of this disease. Moreover, the prevalence of multidrug-resistant TB (MDR-TB) as high as 26.8% has been recently reported [2]. TB is a chronic infectious disease caused by the tubercle bacillus, which is characterized by its slow growth, dormancy and intracellular pathogenesis. It is suggested that *Mycobacterium tuberculosis* (MTB) is a recent pathogen dating back approximately 15,000 years [3]. It is a Gram-positive bacterium and its genome comprises about 4.4 megabase pairs. MTB is also an acid-fast organism which contains large amounts of mycolic acids within their cell walls [4]. These substances resist Ziehl-Neelsen staining and showed a bright red color after staining. Subsequently, the mechanism underlying the loss of acid-fastness in MTB was found to be associated with accumulation of triacylglycerol-containing intracellular inclusions [5].

Aiming at a better understanding of the virulence and immunity in MTB, the complete genome of a strain, H37Rv [6], have been sequenced. Among the approximate 4000 genes in the MTB genome, nearly 25% of them are annotated as hypothetical proteins (HPs), which are encoded by predicted open reading frames but do not have any confirmed functions. In many species, HPs can play important roles in the survival of pathogens and the progression of associated infectious diseases [7, 8]. In MTB, some of these HPs have been experimentally characterized, e.g. Rv0079, which was found to be a DosR regulon playing an inhibitory role in protein synthesis and interacting with TLR2 to promote cytokine secretion [9, 10]. Another example is Rv3873, which was identified to be a PE/PPE family protein that may play crucial roles in the MTB survival in different environments [11]. These previous results indicated that HPs could also play important roles in MTB. However, the functions of most HPs in MTB are still unclear. In this study, we aim at annotating MTB HPs using our recently developed annotation pipeline and the results we present should be helpful for the further characterization of those potentially important HPs.

Several studies have been previously attempted to investigate the function roles of HPs in MTB. Mazandu et al. have predicted the function of MTB HPs using the network topology similarity of gene ontology (GO) term between different species [12]. Doerks et al. have analyzed the function of MTB hypothetical proteomes by the genomic context method [13]. Nevertheless, these studies can only assign rough family information to HPs but not indicate the probable protein homologs. Gazi et al. have investigated the function and structure of 98 conserved HPs by a set of database searching [14]. However, this effort on the annotation of HPs in MTB was mainly focused on assigning functions using protein sequence alignment. Such approaches usually cannot pick up too many homologs for functional characterization. Recently, we have developed a new package called SSEalign for homology identification of HPs using secondary structure element alignment and functional parameters validation [15]. Our SSEalign has shown satisfactory performance for identifying homology of those uncharacterized proteins in minimal bacterial genome JCVI-syn3.0 [16].

In this study, we have investigated the sequence similarity between different species and applied SSEalign to annotate those HPs in MTB. We then execute function enrichment for these annotated proteins and identify several important groups of proteins in MTB. This assignment of protein homologs to MTB HPs should broaden our understanding of their function and provide insights for their future characterization.

## Results and discussion

### Sequence similarity between MTB and other bacteria

**Fig. 1:**
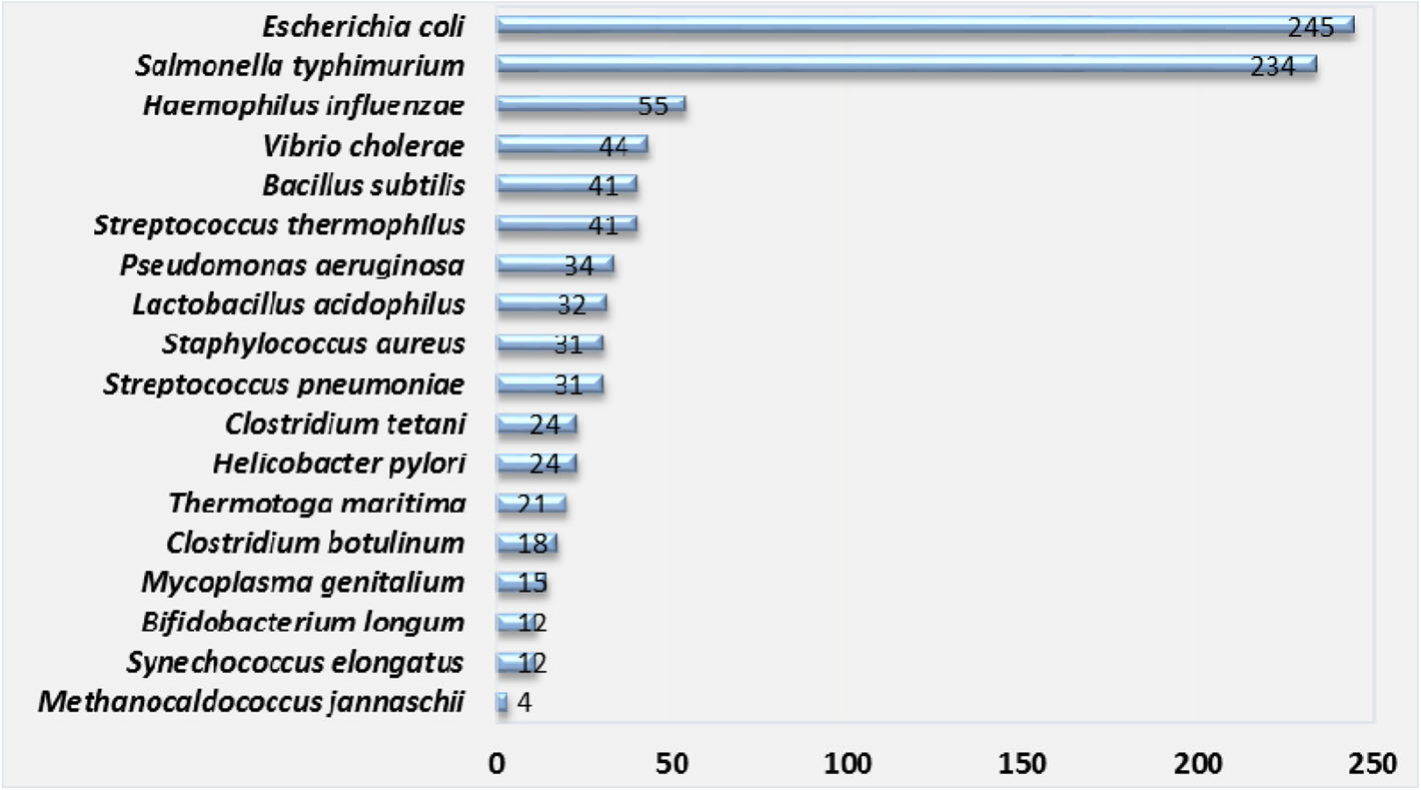
The number of best hits in different species. The numbers indicated the similarity between MTB HPs and the proteins in other species

The 3906 coding proteins of MTB genome could be divided into 1051 HPs and 2855 proteins with known functions (non-HPs). To better annotate these HPs and not be misled by unrelated species, we need to identify the best bacteria for homology identification by SSEalign. The coding sequences of these HPs was aligned against the proteins in other bacteria via BLAST with cutoff E-value = 1e-5. The number of best hits in different species were calculated. We found that the MTB HPs can be found with 245 and 234 best hits in *E. coli* and *S. typhimurium*, respectively (Fig. 1). The numbers of best hits in *E. coli* and *S. typhimurium* were distinctly larger than those in other species, indicating these two bacteria were very suitable for annotation of MTB HPs by SSEalign.

### Homology identification of MTB HPs

**Fig. 2:**
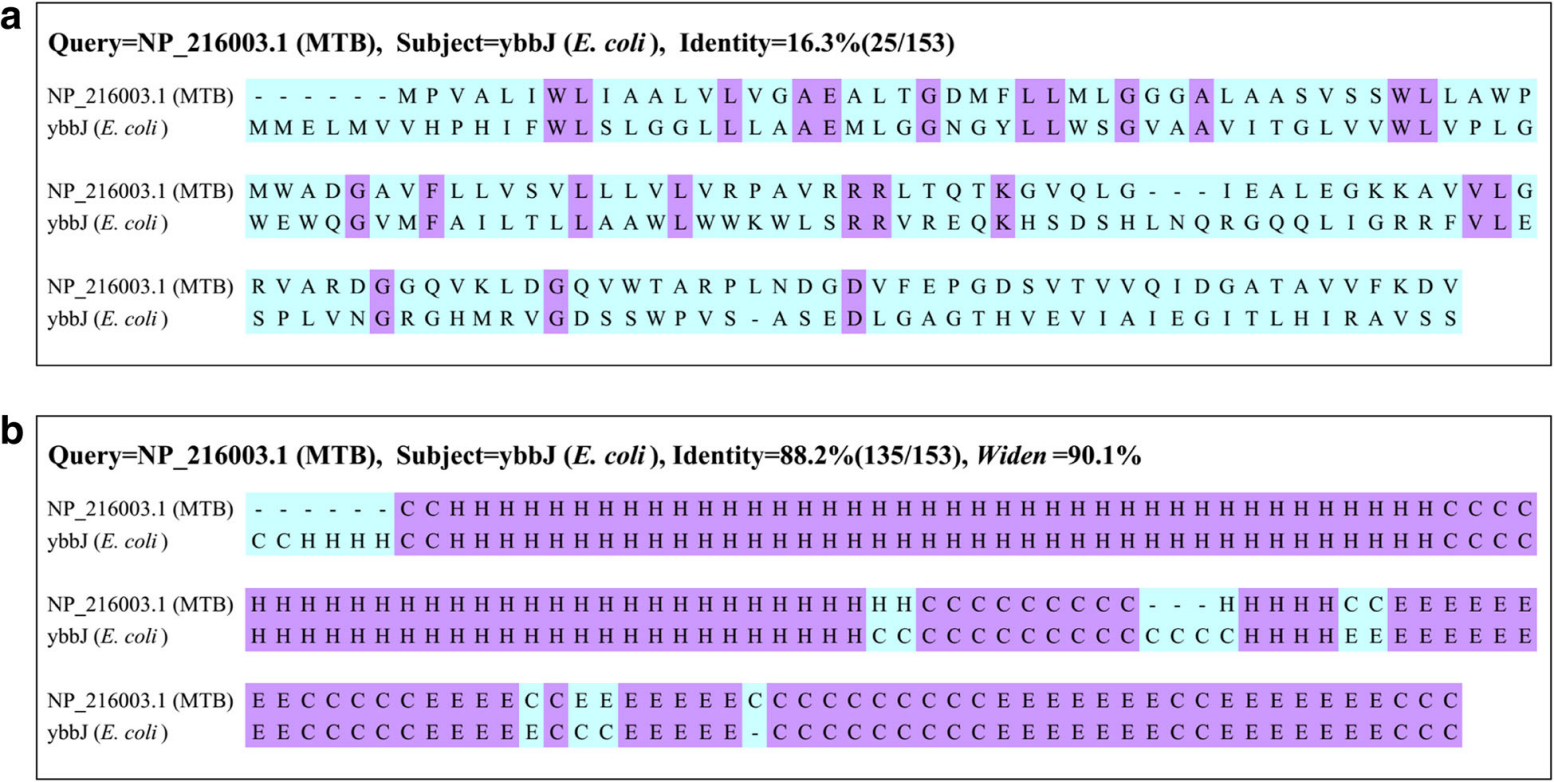
The primary sequence and secondary structure element alignment of MTB NP_216003.1 and *E. coli* ybbJ. **a** The primary sequence alignment of NP_216003.1 and ybbJ; **b** The secondary structure element alignment (SSEalign) of NP_216003.1 and ybbJ

We then identified homologs of the MTB HPs in two bacteria (*E. coli* and *S. typhimurium*) using the SSEalign. Homologs with an FDR cutoff≤0.01 were selected as homology candidates for further evaluation. The performance of SSEalign is satisfactory because homology candidates could be found in 78.3% (823/1051) of the HPs (Additional file 1: Table S1). Compared to previous studies, the functions of some HPs were uniquely identified with high confidence in this study (Table 1**)**. Among these proteins, most of them were supported by at least three parameters, for example, NP_216003.1. The NP_216003.1 shared extremely high *Widen* value (90.1%) and high identity (88.2%), with *E. coli* membrane protein ybbJ by SSEalign, while only an identity of 16.3% could be obtained by their primary sequence alignment (Fig. 2). The same Pfam domain PF01957 was found in both MTB NP_216003.1 and *E. coli* ybbJ. Moreover, these two proteins share consistent interaction with lysine tRNA synthetase lysS in both MTB and *E. coli*. These evaluation results further suggested that our annotation pipeline for HPs in MTB by SSEalign was very convincing.

**Table 1:** Top ten uniquely identified HPs in this study

No.	ORF	Accession number	Annotated gene	Description	*Widen* value %	Supporting number
1	Rv0566c	NP_215080.1	yajQ	Protein YajQ	94.15	3
2	Rv0190	NP_214704.1	rcnR	Transcriptional repressor RcnR	92.89	4
3	Rv0587	NP_215101.1	yciC	Membrane protein YciC	91.12	3
4	Rv2377c	NP_216893.1	ybdZ	Enterobactin biosynthesis protein YbdZ	90.66	5
5	Rv1487	NP_216003.1	ybbJ	Membrane protein YbbJ	90.10	6
6	Rv0025	NP_214539.1	yqjE	Membrane protein YqjE	89.70	3
7	Rv1766	NP_216282.2	holE	DNA polymerase III subunit theta	88.45	4
8	Rv0464c	NP_214978.1	mtlR	Mannitol operon repressor	88.30	3
9	Rv0225	NP_214739.1	rfaB	Lipopolysaccharide 1,6-galactosyltransferase	88.08	4
10	Rv1139c	NP_215655.1	cybB	Cytochrome b561	86.49	3

Compare to Doerks et al.’s and Gazi et al.’s studies, these proteins were uniquely identified in our study

To obtain annotation information from other MTB strains, these HPs were searched against the genomes of nine MTB strains (africanum, BCG, CAS, H37Ra, Haarlem, microti, NITR202, OV254, pinnipedii). Among the 823 HPs annotated in this study, 67 of them could be annotated by other MTB strains (Additional file 1: Table S2). Most of these proteins were known as house-keeping genes, such as the ribosomal RNA rsmG and chaperone protein DnaK.

### Supporting evidence of our annotation

**Fig. 3:**
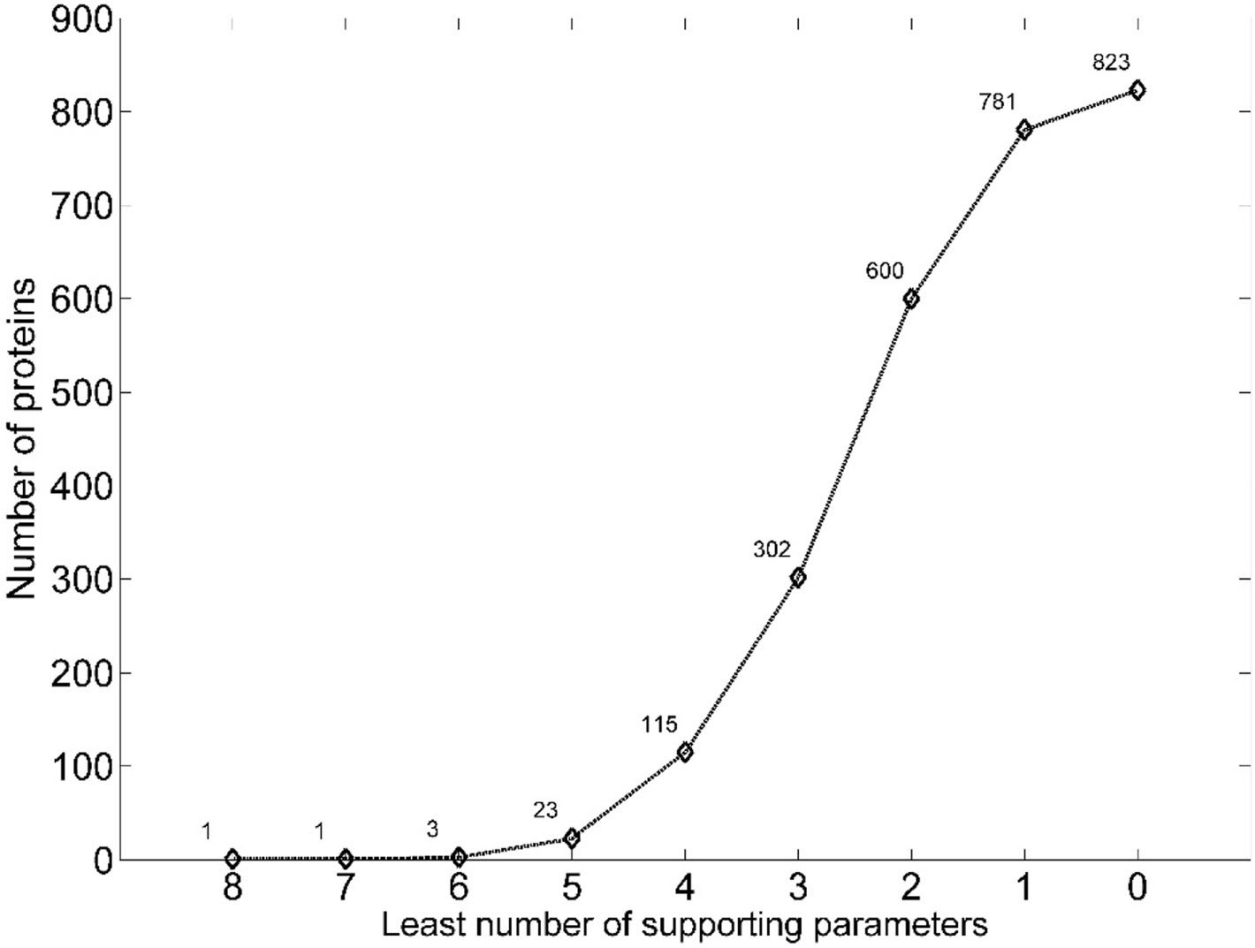
Cumulative distribution of supporting parameters in MTB. The x-axis indicated the cumulative supporting number out of totally eight parameters. The y-axis indicates the number of proteins supported by at least number of parameters in the corresponding x-axis

The identified homologs were further evaluated using eight parameters (Transcriptional evidence, Protein domain, Gene synteny, Protein-protein interaction, Homology modeling, Proline residue distribution, Hydrophobicity profile and Charge distribution) to validate the reliability of our method. Such parameters are independent functional supports of homology pairs identified by SSEalign. We found that 36.7% (302/823) of homologous proteins were supported by at least three parameters and 14.0% (115/823) of them were even supported by four or more parameters (Fig. 3). The result suggests that our annotation of HPs is very reliable.

### Compare with other studies

Previously, Mao et al. [17] reviewed the annotation results of MTB genomes and successfully assigned all MTB proteins into 704 function categories based on structure similarity comparison by Phyre2 [18]. Among them, the protein groups of 473 HPs were assigned and most of the protein group assignments are consistent with our results (Additional file 1: Table S1). The major methodological difference of our work and Mao et al. study is that they applied the three-dimensional structures of known functions to serve as templates while we use the predicted secondary structure for comparison. In addition, their results could only assign HPs to structural groups, but our results could annotate HPs to individual functional proteins. Therefore, our method has a higher precision when compare with Mao et al. study and can facilitate future functional characterization of the annotated HPs. When compared with Mao et al. study, a set of 350 HPs is uniquely annotated in this study.

**Fig. 4:**
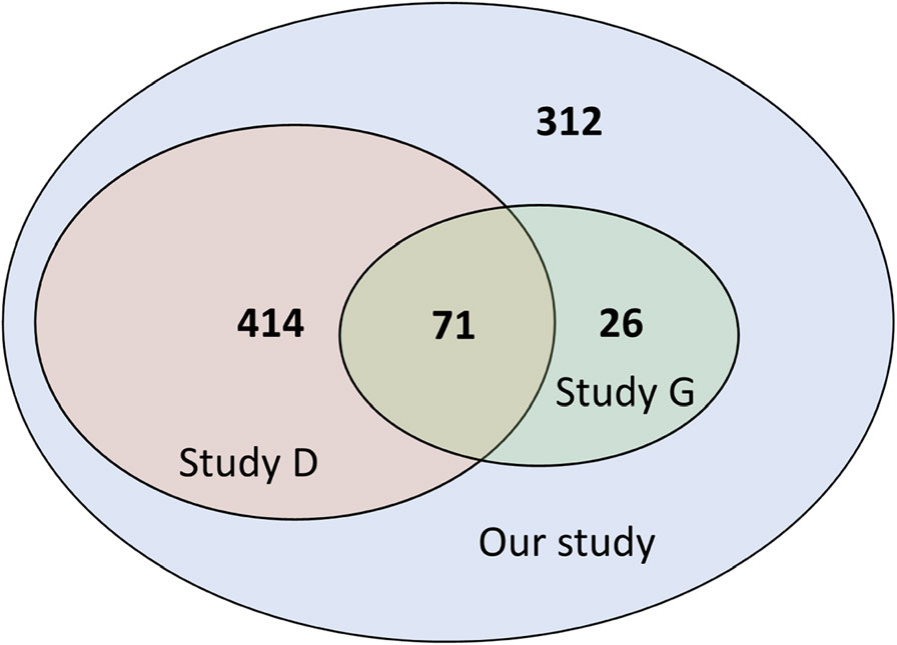
Venn diagram of comparison with other studies. Study D: Doerks et al’s study; Study G: Gazi et al’s study; The number indicated the annotated HP number in each study

Gazi et al. have applied a list of bioinformatics tools, such as ProtoParam and CDD-BLAST to successfully annotate 97 conserved HPs [14]. However, Gazi et al.’s study only focused on the selected 99 conserved HPs, whose research scope is much smaller than our study. Doerks et al. have previously annotated 485 MTB HPs by the combined analysis of automatically generated functional hints form eggNOG ortholog framework [19] and genomic context methods [13]. Nevertheless, Doerks et al.’s annotation of MTB HPs is not specific than this study, for example, 29 HPs were annotated as a uniform description “membrane associated process”. This deficiency in Doerks et al.’s results was probably caused by the use of eggNOG group annotation as their basis of analysis platform. Most of our annotation is consistent with Gazi et al.’s and Doerks et al.’s annotation, while some of the proteins are uniquely identified in our study. By a Venn diagram shown in Fig. 4, we found that 71 proteins could be identified by all three studies. Furthermore, 312 proteins could be annotated by SSEalign but could not be identified by BLAST searching in Gazi et al.’s and Doerks et al.’s studies, such as the above-mentioned protein ybbJ. This protein appears to be highly diverse in different bacteria (Fig. 2), leading to the failure in annotations by sequence alignment approach. However, the secondary structures of these proteins are extremely conserved during the evolution, explaining why SSEalign has such an excellent performance for the annotation of these proteins.

### Function enrichment for identified proteins

**Fig. 5:**
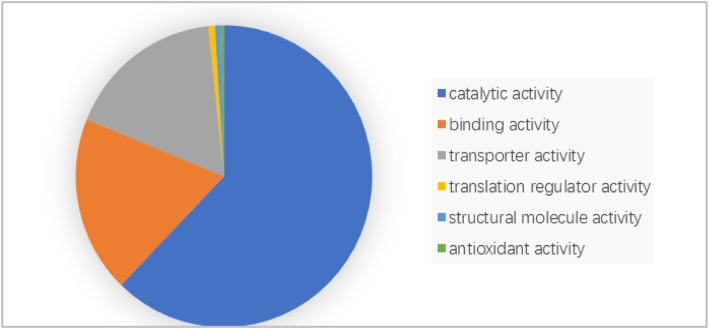
Categories of annotated MTB hypothetical proteins. The largest proportion of annotated proteins was found in the category of “catalytic activity”

We then assigned these 823 proteins into different functional categories by PANTHER classification analysis. Clustering of these 823 proteins showed that the top categories were proteins with catalytic activity and proteins with binding activity, which constituted 62% (512/823) and 19% (156/823), respectively (Fig. 5). The proteins with transporter activity were also relatively high by a ratio of 17%. As an intracellular pathogen, MTB can be inhaled into the alveoli from the air and subsequently engulfed by alveolar macrophages. There are many receptors on the surface of macrophages that can recognize pathogen and protect the host cell. To survival in host macrophages, MTB requires a large number of transporters to absorb essential metabolites and excrete toxic materials. Pathogen such as MTB could transport out virulence factor and utilize these specific receptors in macrophage membrane to invade into host cells [20]. This result explains why MTB have so many transporters in the genome.

### Novel virulence factors and antigen proteins

Virulence factors are proteins produced by pathogenic bacteria which bring undesirable damage to the host. The VICMpred and VFDB databases were used to predict potential virulent factors from MTB HPs. Twelve annotated HPs were found in VFDB database and were predicted by VICMpred with consistent results (Table 2). These virulent factors, such as RNA polymerase sigma factor rpoS and acetyltransferase epsM, are promising target candidates for the treatment of MTB infection and could act as an adjunct molecule for the host-pathogen interaction. The rpoS is a subunit of RNA polymerases that recognize the promoter regions of the genes. In host environment, MTB are constantly assaulted by a variety of stresses that include nutritional deprivation and DNA-damaging agents. To survive such challenge, bacteria usually evolved the coordinated cellular defense mechanisms such as the SOS response to DNA damage and rpoS-regulated response to nutritional deprivation [21]. Previously, only SOS response was reported in MTB, while rpoS-regulated response has not been reported [22]. We suggest that it could be highly possible due to the failure annotation of protein rpoS. Moreover, ten annotated proteins were predicted to be potential antigen proteins by searching against the MtbVeb and IEDB databases (Table 2). For example, the protein pspA was predicted to be an antigen protein that could be used to develop a vaccine for treating MTB infection. This protein is a phage shock protein that helps cells avoid the impact of agent impairing cell membrane function and maintenance of the protonmotive force under stress conditions [23]. It has been reported that pspA was involved in divalent metal transport and is required for virulence in *Salmonella typhimurium*, suggesting that pspA could be a vaccine candidate for future investigation [24].

**Table 2:** Identified virulence factors and antigen proteins in MTB

ORF	Accession number	Annotated gene	Our annotation	Antigen protein
Rv2707	NP_217223.1	rpoS	RNA polymerase sigma factor rpoS	NO
Rv0674	NP_215188.1	pspA	Phage shock protein pspA	YES
Rv1518	NP_216034.1	wcaA	Colanic acid biosynthesis glycosyl transferase wcaA	YES
Rv0498	NP_215012.1	tagE	alpha-glucosyltransferase	YES
Rv1893	NP_216409.1	prm1	Plasma membrane fusion protein prm1	YES
Rv0546c	NP_215060.1	gloA	Lactoylglutathione lyase	YES
Rv1505c	NP_216021.1	epsM	acetyltransferase epsM	YES
Rv0049	NP_214563.1	ygaZ	Inner membrane protein ygaZ	YES
Rv2694c	NP_217210.1	artP	Arginine transport ATP-binding protein artP	NO
Rv2365c	NP_216881.1	ydbL	Protein ydbL	YES
Rv2327	NP_216843.1	slyA	Transcriptional regulator slyA	YES
Rv2751	NP_217267.1	yktD	adenosyl-methionine-dependent methyltransferase yktD	YES

Twelve new virulence factors were found in MTB in this study. The antigen proteins could be applied to vaccine development for the prevention of MTB

### Drug target candidates

An ideal drug target is a kind of essential protein with pathogen-specific characteristics. It required that no close homolog of this bacterial protein was present in the human proteome to minimize the risk of undesirable side effect. Among the 823 annotated proteins, 53 of them were identified as essential proteins by searching against DEG database. A strict host non-homologous analysis was carried out to identify those proteins with no homologs in human proteome. Out of 53 proteins, 30 did not show any significant hits with an E-value threshold of 10. A further analysis was carried out to assess the druggability of the shortlisted 30 candidate proteins. Among these proteins, six of them were found to be druggable by chEMBL target searching (Table 3), such as Rv0817 and Rv2927c. Laminin hydrolytic enzyme (Rv0817) is a well-known drug target and ocriplasmin could be used to inhibit the activity of this protein for treating bacterial infection. Rv2927c is a kind of ATP synthase and was previously reported as a drug target of bedaquiline fumarate. The rest of putative drug target candidates can be also considered as novel targets, which should be further validated experimentally.

**Table 3:** Potential drug targets of MTB HPs

ORF	protein ID	ChEMBL ID	Drug name	Mechanism of Action
Rv0817c	NP_215332.1	CHEMBL2095222	ocriplasmin	Laminin hydrolytic enzyme inhibitor
Rv2927c	NP_217443.1	CHEMBL2105700	bedaquiline fumarate	ATP synthase inhibitor
Rv1303	NP_215819.1	CHEMBL3989689	squalamine	Sodium/hydrogen exchanger 3 inhibitor
Rv2908c	NP_217424.1	CHEMBL564085	troleandomycin	70S ribosome inhibitor
Rv2926c	NP_217442.1	CHEMBL614	pyrazinamide	Fatty acid synthase inhibitor
Rv0312	NP_214826.1	CHEMBL1201780	carglumic acid	Carbamoyl-phosphate synthase inhibitor

Six essential HPs were identified with potential drug targets

## Conclusions

MTB genome has been studied for several decades by scientists. However, the function of approximately one quarter of the protein is unknown in the annotation version of H37Rv. In this study, we have applied a series of comparative genomics tools including a newly developed tool SSEalign to investigate functional role of HPs in MTB. A set of 823 proteins were annotated and most of them were supported by at least two independent parameters. We found a high proportion of HPs belonging to proteins with catalytic activity, indicating that lots of enzymes were ignored in un-annotated proteins. We found the members of transporters are also relatively high, suggesting the possible mechanism of frequent absorbing essential metabolites and excreting toxic materials in MTB. Compared to previous study, our annotated protein number is significantly larger than Gazi et al.’s and Doerks et al.’s studies. Twelve virulence factors and ten vaccine candidates, including two critical genes (rpoS and pspA) involved in the stress response pathway in MTB, were identified in HPs. Our finding serves as new clues for treatment of TB caused by MTB.

## Methods

### Genome and proteome

**Fig. 6:**
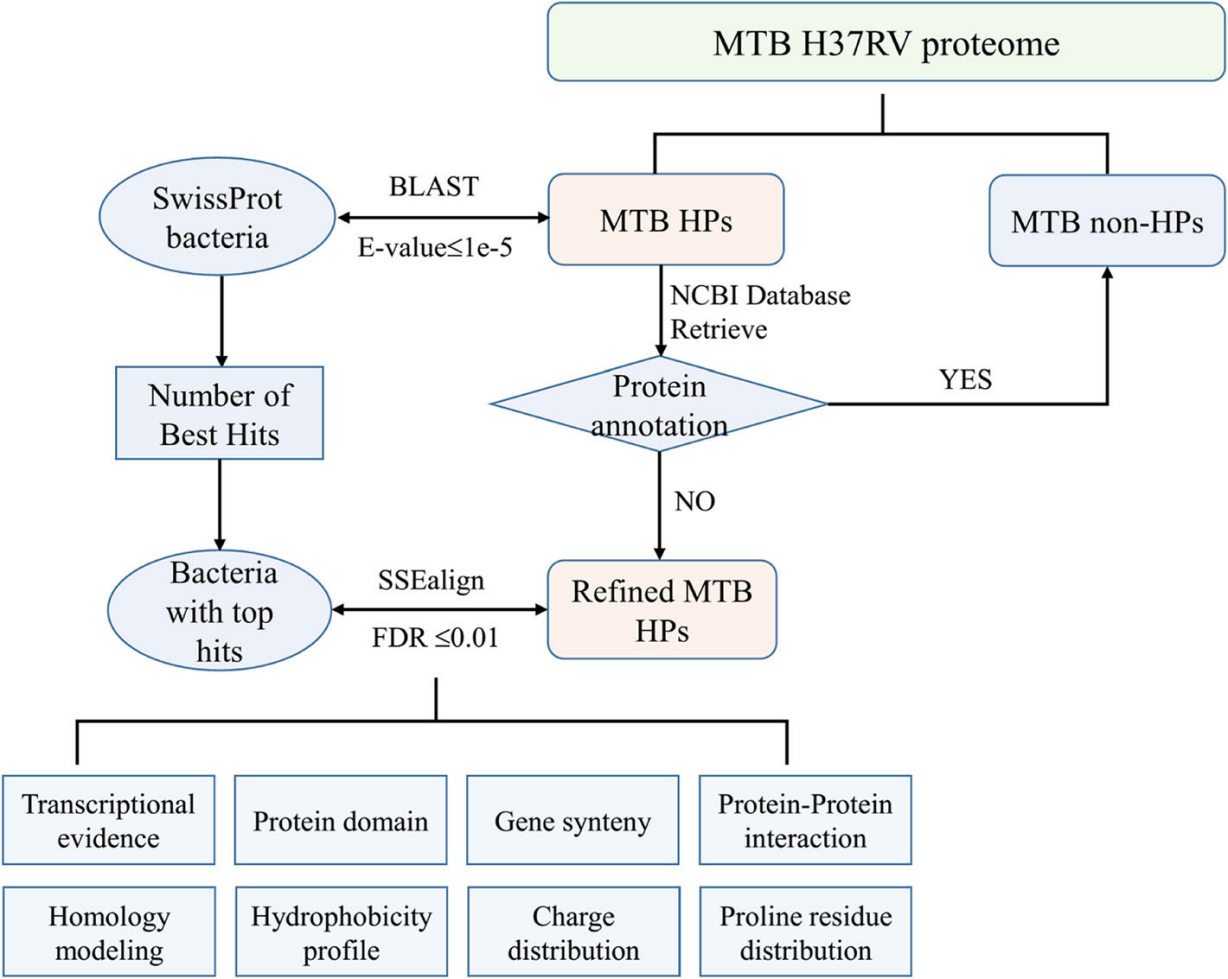
The workflow for the annotation of MTB HPs. HP: hypothetical protein; FDR: false discovery rate; SSEalign: a method for homology identification

The annotated genome of MTB H37Rv was downloaded in NCBI Genome database [25]. The coding proteins with annotation of “hypothetical protein” or “uncharacterized protein” were picked out and denoted as HPs. Those proteins with detailed annotation were denoted as non-HPs. The gene expression data of MTB were downloaded from TB database [26]. The whole datasets of bacteria proteomes were retrieved from UniProt database [27]. The flowchart of our work is shown in Fig. 6.

### Sequence similarity searching

Before we applied SSEalign to identify homologs of MTB HPs, we need to select the best candidate species as the reference species. The MTB HPs were searching in the database of bacterial genomes by BLAST [28] with a cutoff E-value≤1e-5. The numbers of best hits in each bacterium were then calculated. Those species with the greatest number of top hits were selected as candidate species for homology identification by SSEalign. As the genome annotations of other MTB strains may provide using information about H37Rv HPs, we had also searched the H37Rv genome against other published genomes of MTB strains so as to cross-check the annotation of H37Rv HPs in these genomes.

### Homology identification of MTB HPs

SSEalign is a package for homology identification based on secondary structure element alignment developed by our group. It showed satisfactory performance in the annotation of the bacterial minimal genome JCVI-syn3.0. Thus, in this study we employed this package to annotate the MTB HPs protein. Bacteria with the greatest numbers of top hits were selected as the candidate bacteria for homology identification by SSEalign. The Widen (Weighted identity) value was used as a cut-off for secondary structure similarity. In our previous study [15], the relationship between the Widen value and FDR (False discovery rate) has been reported. Each Widen value could generate an FDR value by the Bootstrap strategy. The threshold was set as FDR ≤ 1%. For each HP, the homolog is selected for further validation if its homologs identified were consistent with each other.

### Supporting evidence of our annotation

The quality of protein function annotation can be measured in a computational way. The following eight parameters served as the supporting evidence of our annotation result.

- Transcriptional evidence: Because the coding proteins were just predicted by software automatically, some proteins could even not be transcribed in MTB. To remove those false positives, we downloaded the gene expression data from TB database [26]. If the proteins showed expression in MTB, these results were considered supported in the parameter of transcriptional evidence.
- Protein domain: The protein domains of HPs and target protein were predicted by InterProScan [29] and Pfam [30] databases. We considered that our results were supported by the parameter of protein domain if the two homologous proteins share the same domains.
- Gene synteny: It has been reported that the homologs commonly share the gene synteny among different species [31]. Thus, the neighboring genes in upstream and downstream were compared in MTB and target species. We considered that our results were supported by the parameter of gene synteny if the homologs share the corresponding gene synteny.
- Protein-protein interaction (PPI): The homologs commonly share the same protein-protein interactions in different species. The protein-protein interaction datasets of *E. coli* and MTB were obtained from BioGrid database [32]. If the HP and the annotated protein could interact with the same protein in both *E. coli* and MTB, this homologous pair was considered supported in the parameter of protein-protein interaction.
- Homology modeling: We hypothesize that homologous proteins tend to have similar tertiary structures. If tertiary structures of annotated proteins are available, the structure of HPs was predicted by SWISS-MODEL [33]. The quality of the generated protein models was evaluated by their backbone conformations, the placement of core side chain and threading angle. If these homologous pairs have satisfactory accurate protein models generated by homology modeling, they were considered as supported by the parameter of homology modeling.
- Hydrophobicity profile: We hypothesize that homologous proteins tend to have similar hydrophobicity profile. The MTB HP and candidate homologous proteins were separated into ten ordered blocks of the same size according to the primary sequence. The hydrophobicity profile of these proteins was predicted by EMBOSS [34]. The average hydrophobicity values of the blocks of two protein were enumerated and the significance was assessed by pairwise Z-test. If the proteins showed significant *p*-value (≤0.05) comparing their 10-block hydrophobicity value, these homologous pairs were considered supported in the parameter of hydrophobicity profile.
- Charge distribution: We hypothesize that homologous proteins tend to have similar charge distribution. The MTB HP and candidate homologous proteins were also separated into ordered ten blocks of the same size according to the primary sequence. The charge distribution of these proteins was predicted by EMBOSS. The average charge values of the blocks of two protein were enumerated and the significance was assessed by pairwise Z-test. If the proteins showed significant *p*-value comparing their 10-block charge value, these homologous pairs were considered supported in the parameter of charge distribution.
- Proline residue distribution: Since proline residues in protein structures have been found to play important structural roles in guiding protein folding [35], we hypothesize that homologous proteins tend to have a similar distribution of proline residue. We investigated the proline residue distribution in primary and secondary sequences of homologous proteins. If the proline residue located in the transformation site of alpha helix and beta sheet, these results were considered supported in the parameter of proline residue distribution.

### Function enrichment for identified proteins

We conduct function enrichment of annotated proteins to better show the hierarchy structure of our annotation. The PANTER database contains comprehensive information on the function of various proteins extracted from hundreds of completely sequenced genomes. Thus, PANTHER tools were used to identify the functional categories of annotated proteins [36]. Furthermore, the pathways of these annotated proteins were analyzed by the KEGG database [37].

### Potential virulence factors and antigen proteins

The virulence factors are produced by pathogenic bacteria and viruses that bring undesirable damage to the host. In this study, the virulence factors were predicted by VirulentPred [38], which use cascade SVM algorithm to identify virulence factors based on the amino acid dipeptide composition. We also obtain a list of the known virulence factors of pathogenic bacteria from VFDB database [39] and inferred their homologs in MTB*.* Regarding the strong pathogenicity of MTB, it is also a major concern to develop the potential vaccine for the treatment of MTB infection. To identify the potential antigen proteins within those annotated HPs, we searched the annotated HPs against the MtbVeb database [40] and the Immune Epitope Database (IEDB) [41]. These antigen proteins could be used as vaccine candidates in future studies.

### Potential drug target

A desirable drug target is a kind of essential proteins with no homology in human proteome. The essential proteins among MTB HPs were picked out based on a list of essential genes retrieving from Database of Essential Genes (DEG) [42]. All annotated essential proteins were subjected to a search against the non-redundant database of human proteome with a cutoff E-value = 1e-5. Protein sequences that showed no significant hits were retained for further analysis. The shortlisted proteins were then searched against chEMBL [43] and Drugbank [44] database to find if any suitable drug were present for this target. The presence of non-homologous proteins in these two databases with the same function could serve as a piece of strong evidence for their druggable property. As these proteins have not been reported in MTB, they can be used as novel drug target candidates for future study.

## Additional file

Additional file 1: **Table S1.** The complete list of annotated MTB HPs. **Table S2.** The 67 annotated proteins identified by searching against genomes of other MTB strains. (XLSX 141 kb)

## Abbreviations

DEG: Database of essential genes
FDR: False discovery rate
HP: Hypothetical protein
MDR-TB: Multidrug-resistant TB
MTB: *Mycobacterium tuberculosis*
PPI: Protein-protein interaction
SSEalign: Secondary Structure Element alignment
TB: Tuberculosis
VFDB: Virulent factor database
WHO: World Health Organization
*Widen*: Weight Identity, a score to evaluate similarity in package SSEalign
